# Recent Progress of Toxic Gas Sensors Based on 3D Graphene Frameworks

**DOI:** 10.3390/s21103386

**Published:** 2021-05-13

**Authors:** Qichao Dong, Min Xiao, Zengyong Chu, Guochen Li, Ye Zhang

**Affiliations:** College of Liberal Arts and Science, National University of Defense Technology, Changsha 410073, China; qichao_dong@163.com (Q.D.); a2805173694@163.com (M.X.); lgc_nudt@126.com (G.L.); zhangye8905@foxmail.com (Y.Z.)

**Keywords:** graphene, graphene hydrogel, graphene aerogel, gas sensor

## Abstract

Air pollution is becoming an increasingly important global issue. Toxic gases such as ammonia, nitrogen dioxide, and volatile organic compounds (VOCs) like phenol are very common air pollutants. To date, various sensing methods have been proposed to detect these toxic gases. Researchers are trying their best to build sensors with the lowest detection limit, the highest sensitivity, and the best selectivity. As a 2D material, graphene is very sensitive to many gases and so can be used for gas sensors. Recent studies have shown that graphene with a 3D structure can increase the gas sensitivity of the sensors. The limit of detection (LOD) of the sensors can be upgraded from ppm level to several ppb level. In this review, the recent progress of the gas sensors based on 3D graphene frameworks in the detection of harmful gases is summarized and discussed.

## 1. Introduction

There is a huge demand for the development of simple and reliable gas sensors [1]. In many fields, such as agriculture, medical diagnosis, and industrial waste, especially in environmental monitoring, it is necessary to detect NO_x_ (especially NO_2_), ammonia (NH_3_), and volatile organic compounds (VOCs), because of their possible toxicity and related risks to the ecosystem [2,3]. In many countries, air pollution is a major environmental problem caused by rapid industrialization. A large amount of NO_2_ is emitted into the environment every year due to the industrial combustions and automobile emissions [4]. Therefore, the detection of NO_2_ has aroused widespread concerns, because it is harmful to the plants and respiratory systems of people and animals [5]. Additionally, NO_2_ can cause acid rain and photochemical smog [6,7]. Therefore, the United States Environmental Protection Agency (EPA) defines NO_2_ as a typical air pollutant, and the exposure limit is only 53 ppb [8]. Ammonia (NH_3_) is also a common dangerous air pollutant, which is produced by the industrial process, agricultural production, and manufacturing process [9,10]. Specifically, any overexposure to the high concentrations of NH_3_ (>30 ppm, 10 min) can irritate the human eye, skin, and respiratory system [11,12,13]. VOCs are the hydrocarbons that exist as gases or vapor at room temperature, which can be emitted from numerous products and activities, e.g., detergents, paints, solvents, tools, clothes, toys, cleaning, and cooking [14]. Aldehyde, aromatic, aliphatic, halogenated, and terpenoid compounds are the VOCs commonly detected in commercial buildings [14,15]. Toxic VOCs that have been previously detected in air by any type of sensors include formaldehyde, acetaldehyde, benzene, toluene, xylenes, phenol, pyridine, acetone, acetic anhydride, carbon disulfide, dihydroxybenzene, and so on [14,15,16,17,18,19]. For example, phenol is a toxic VOC occurring both naturally but also from industrial processes, which can be rapidly absorbed through the skin and cause skin and eye burns upon contact [20]. It is considered as a serious pollutant because of the toxicity and persistence in the environment. The short-term exposure limit of phenol is 10 ppm, 60 min [21]. Because of the serious environmental pollution, phenol monitoring becomes an urgent problem. Therefore, with the monitoring development of air pollution, the demand for gas sensors will increase rapidly in the future.

As a 2D material, graphene has many advantages, such as large conjugated structure, high specific surface area, high conductivity, easy to be synthesized, sensitive to the gas molecules, and so on. It has been proven to be a promising high-performance gas detection material [22]. Graphene surface can easily absorb some molecules, such as NO_2_, NH_3_, CO_2_, and so on. Moreover, the conductivity of graphene will change after adsorption of target gas molecules. The concentration of target gas in the environment can be detected by monitoring the change of conductivity. There have been many reports on the application of graphene in gas sensors, including pure graphene [23,24,25,26] and graphene composite materials [27,28,29,30,31]. There are many factors affecting graphene-based sensors, including: synthetic method [32,33,34], chemical structure [35,36,37], interlaminar structure [34,38], testing environment [39,40,41,42], and surface properties [43,44,45,46,47]. Due to the *π-π* accumulation and Van Der Waals force binding between graphene, the 2D graphene nanocomposites tend to agglomerate, resulting in the reduction of specific surface area [48,49,50]. In order to make full use of the characteristics of graphene, 2D graphene is usually assembled into a three-dimensional (3D) framework state by a series of methods. In contrast, due to the combination of 3D porous structure and the inherent characteristics of graphene, 3D graphene provides more space and larger surface area to transport and store electrons. 3D graphene has good conductivity, large specific surface area, and versatile gas adsorption sites. Furthermore, the defects and edge positions on the 3D porous graphene play an important role in promoting gas adsorption [48]. In recent years, compared with 2D graphene structures, 3D porous graphene structures such as graphene hydrogels, graphene aerogels, and graphene foams have been used as high-performance gas sensors [49]. Although 3D graphene has broad prospects in the field of gas sensors with the super high sensitivity, the selectivity is not satisfactory. Different gas molecules may adsorb on the same 3D graphene sheets and lead to the total change of the resistance [50,51]. It is difficult to quantitatively distinguish one target gas from a gas mixture. To improve the selectivity, defect engineering is generally needed to modulate graphene [52].

Several reviews have presented the main development of graphene-based gas sensors. For example, in 2015, Meng et al. [49] reviewed the graphene-based hybrids for chemi-resistive gas sensors. They focused on the sensing principles and synthesis processes of the graphene-based hybrids with noble metals, metal oxides, and conducting polymers. In 2018, Xia et al. [50] summarized the 3D structure graphene/metal oxide hybrids for gas sensors. They concluded a variety of logical strategies to design the 3D nanohybrids of RGO and MOx. In 2020, Ilnicka et al. [51] summarized the graphene-based hydrogen gas sensors, a special case of gas sensitivity to H_2_. However, the above reviews did not reflect the whole progress of graphene gas sensors, especially for the air pollution monitoring applications. This paper aims to summarize the recent progress of the gas sensors based on 3D graphene frameworks in the detection of air pollutants.

## 2. Synthesis of 3D Graphene Frameworks

Graphene oxide (GO) and reduced graphene oxide (RGO) have a 2D conjugated structure with single-atom thickness and residual oxygen-containing groups, which can be regarded as 2D conjugated macromolecules, structurally. They have rich chemical activities, which are helpful for 3D self-assembly through a series of chemical modification methods to regulate the interaction between the layers [34,38].

**Figure 1:**
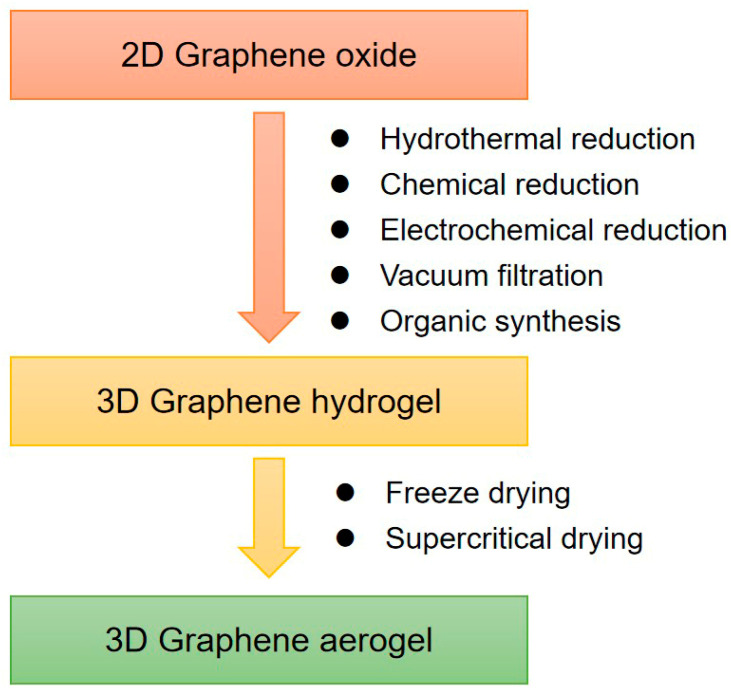
Synthesis methods of 3D graphene frameworks.

Graphene hydrogel is one of the major 3D assemblies. Chemically modified graphene (CMG) hydrogels prepared from GO or RGO can be used for large-scale production. As shown in Figure 1, RGO hydrogels (RGOHs) can be obtained by the following methods:

(1) Hydrothermal reduction, which is simple, fast, and free of impurities. At present, the commonly used hydrothermal method is to prepare RGO dispersion by hydrothermal treatment at 180 °C [53,54,55].
(2) Chemical reduction, which is beneficial for large-scale production, and various reducing agents can be selected [56,57,58,59,60,61,62,63,64,65].
(3) Electrochemical reduction [66,67,68]. The hydrogel prepared by this method is applied to the electrode surface and can be directly applied to the electrode materials of electrochemical instruments.
(4) Vacuum filtration. A simple vacuum filtration method was developed to prepare RGO hydrogels with high conductivity, anisotropy, and responsive stimuli [69,70].

In addition to the 3D self-assembly of graphene in a water system, the assembly of the graphene in an organic system can also be achieved by thermal solvent reduction [71,72,73].

Graphene aerogel composites are usually prepared by supercritical drying or freeze-drying of hydrogel precursors [74,75]. For example, highly compressible RGO aerogels can be obtained by freeze-drying and microwave treatment. Directional freezing is a well-known processing technology of porous materials. This technology can also be used for the preparation of graphene aerogels [76]. Moreover, the controllable heat treatment technology can also reduce GO to RGO and restore conductivity. The regulation of the chemical structure of GO can adjust the morphology and elasticity of aerogels, for example, the oxygen functional groups in GO have a significant effect on the morphology and elasticity of the gels [77].

## 3. NO_2_ Gas Sensors

The development of a highly selective NO_2_ gas sensor with ppb detection limit is an important requirement for continuous environmental monitoring [78] and early diagnosis of respiratory diseases [79]. However, the original graphene, RGO, and RGOH had limited reaction to NO_2_, and could not monitor NO_2_ gas below 100 ppb [80]. Therefore, researchers have explored a variety of methods to fabricate graphene composites. Compared with 2D RGO, 3D RGO is more sensitive to NO_2_ [81]. Table 1 lists the gas sensitivities of 3D graphene toward NO_2_. The response is generally given in the form of relative change of resistance (∆R/R_0_) or conductance (∆G/G_0_), which was tested at a set temperature and a set concentration of NO_2_. S_3D_ and S_2D_ are the sensitivities (responses per ppm) of 3D and 2D graphene, respectively. The graphene with a 3D structure can increase the gas sensitivity to one or two orders of magnitude higher. The LOD of the sensors can be upgraded from ppm level to several ppb level.

### 3.1. 3D MoS_2_/RGO

**Figure 2:**
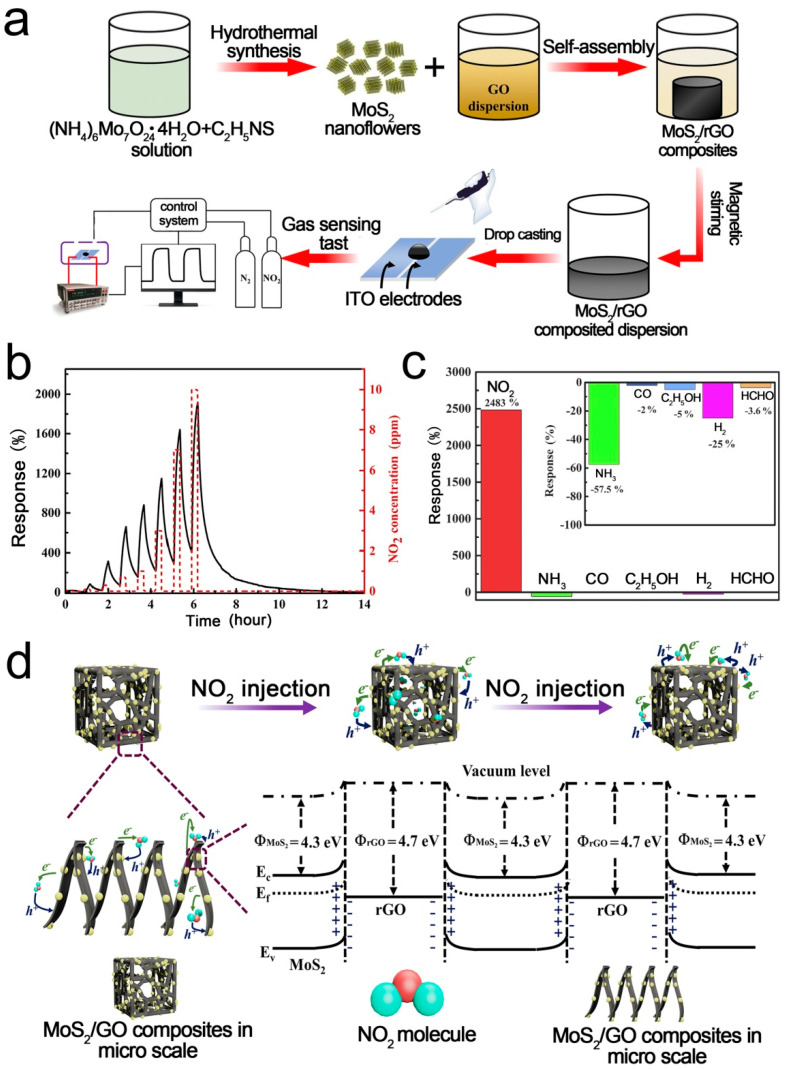
(**a**) Fabrication process of 3D MoS_2_/RGO; (**b**) Dynamic gas responses of MoS_2_/RGO; (**c**) Responses of MoS_2_/RGO-5; (**d**) Schematic illustration of the sensing mechanism [52].

2D-layered MoS_2_ is an excellent gas sensing material due to its high surface/volume ratio and excellent electronic properties [90,91,92,93,94]. However, MoS_2_ nanoparticles tend to agglomerate, which limits their applications. Loading MoS_2_ on 3D RGO is a good choice [95,96,97]. Chen et al. [52] reported a highly sensitive NO_2_ sensor based on 3D MoS_2_/RGO composites. The composites were prepared using a novel self-assembly and hydrothermal method. The mild synthesis process enables MoS_2_ to uniformly disperse on the 3D RGO framework. The agglomeration of MoS_2_ was significantly alleviated, resulting in excellent low-temperature sensing performance. Figure 2 shows the fabrication process, gas responses, and sensing mechanism of the 3D MoS_2_/RGO sensor. The selectivity of the fabricated sensors was characterized using a variety of independent gases. The device shows higher sensing response towards N-based molecules (e.g., NO_2_, NH_3_) than the other gases (CO, C_2_H_5_OH, H_2_, HCHO). When the sensor is exposed to NO_2_ atmosphere, the minority charge carriers (electrons) in the MoS_2_/rGO composites will transfer to NO_2_ due to the strong electron negativity of NO_2_. Thus, the width and height of the heterojunction are reduced, leading to the increased conductivity. A small variation in barrier height and width caused by gas adsorption or desorption can have a significant influence on the resistance. A superior low-temperature NO_2_ sensing performance with a response of 2483% toward 10 ppm NO_2_ was achieved at 80 °C.

### 3.2. 3D SnS_2_/RGO

**Figure 3:**
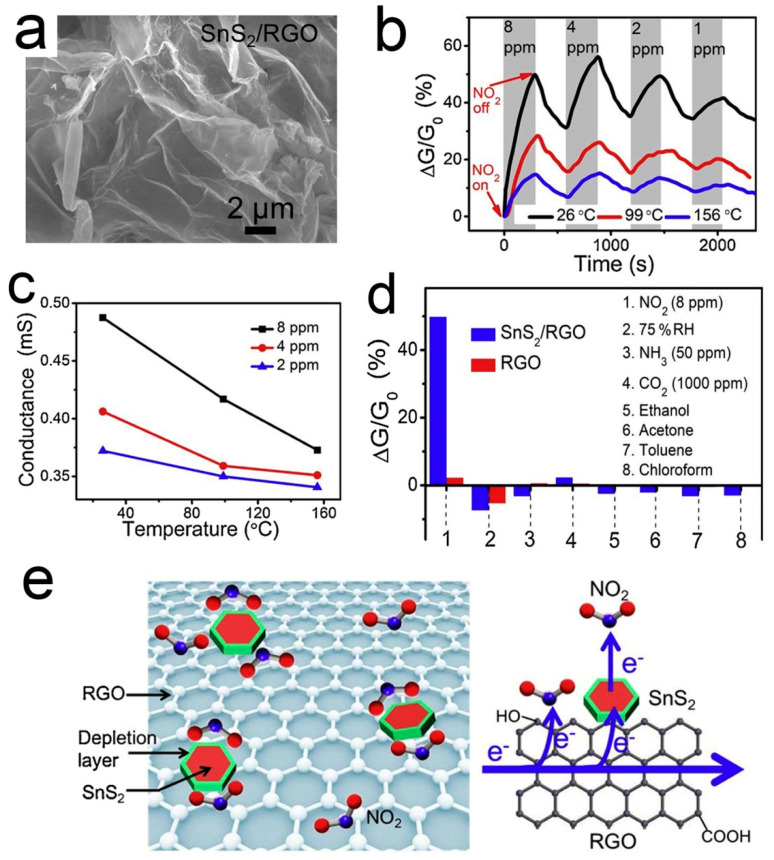
(**a**) SEM image of 3D SnS_2_/RGO; (**b**) Dynamic responses of SnS_2_/RGO to NO_2_; (**c**) Conductance variation of SnS_2_/RGO versus operation temperature; (**d**) RT sensing responses of SnS_2_/RGO and RGO; (**e**) Schematic illustration of the NO_2_ sensing mechanism of the SnS_2_/RGO sensor [86].

Different from *p*-type RGO, SnS_2_ is an *n*-type semiconductor with an indirect band gap of 2.2 ev [98]. SnS_2_ has a high affinity to NO_2_ because of its much weaker electronegativity than NO_2_ [99,100]. However, the resistance of SnS_2_ is too high to be measured at room temperature, so SnS_2_ is not suitable for monitoring NO_2_ at room temperature. When SnS_2_ is coupled with 3D graphene, their properties are complementary to each other. Wu et al. [86] demonstrated a kind of 3D structured SnS_2_/RGO heterojunction, which was synthesized through a facile hydrothermal route. Figure 3 shows the microstructure, gas responses, and sensing mechanism of the SnS_2_/RGO sensor. The 3D structure enhances the adsorption and diffusion of small NO_2_ molecules. SnS_2_ can facilitate the electron transfer from RGO to NO_2_ by forming a heterojunction with RGO. The depletion layer with an electrostatic field at the *p*-*n* heterojunction region could promote the dissociation of NO_2_ and thus enhance the NO_2_ adsorption at low temperatures. Upon NO_2_ adsorption on SnS_2_, the electron will transfer from SnS_2_ to NO_2_, leading to the increase both of the electron-depletion region and the hole concentration of the *p*-type RGO. Thus, the resistance of SnS_2_/RGO is reduced. The sensor displays impressive NO_2_ sensing performance, including high sensitivity (6.1 ppm^−1^), low LOD (8.7 ppb), good linearity, and reversibility.

### 3.3. 3D Eu(TPyP)(Pc)/RGO

**Figure 4:**
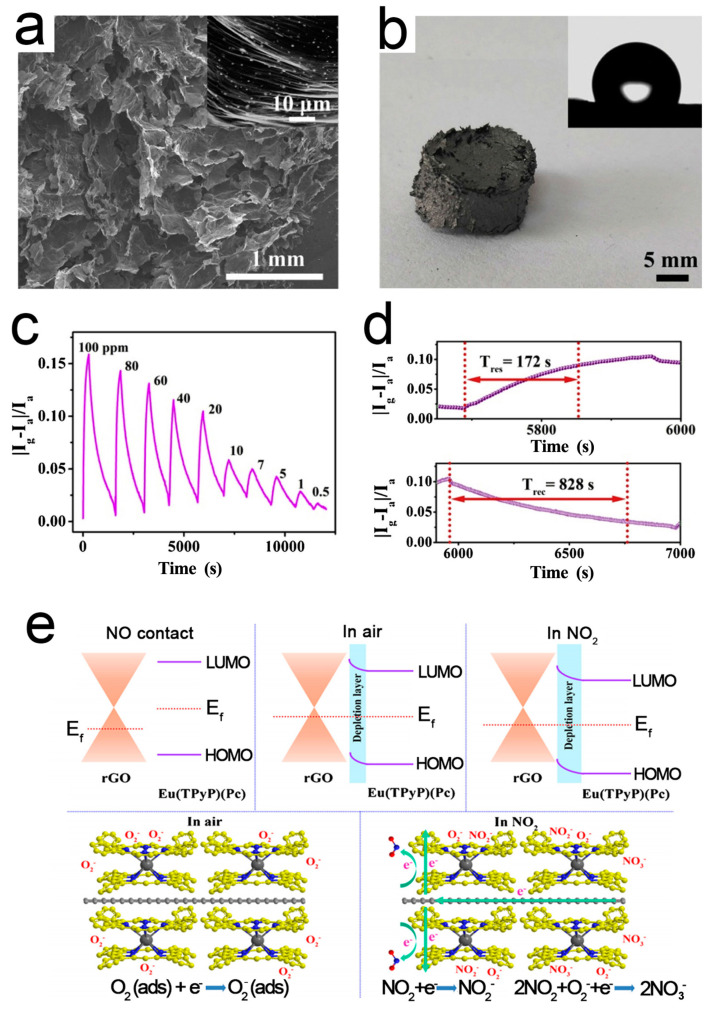
(**a**) SEM image of 3D SnO_2_/RGOH; (**b**) Dynamic responses of RGOH and SnO_2_/RGOH to NO_2_; (**c**) RT responses of SnO_2_/RGOH and RGOH; (**d**) Real-time responses of the flexible SnO_2_/RGOH sensor. Inset: Photograph of the sensor with the bent angle of 150° [85].

Pure RGO sensors have poor selectivity, slow response, and long recovery time, which limits their wide applications [89]. Chemical/physical modification with the external groups or atoms is an effective method [101,102]. Zhu et al. [88] reported a sandwich-type double-decker complex Eu(TPyP)(Pc) (TPyP = meso-tetra(4-pyridyl)porphyrin; Pc = phthalocyanine), which was in situ self-assembled on the surface of RGO driven by the *π–π* interaction, forming a 3D RGO/Eu(TPyP)(Pc) hybrid aerogel. Eu(TPyP)(Pc) not only acts as a sensor recognition unit, but also helps to enhance the amplification effect of the *p*-*n* heterojunction. At the same time, it provides enough space for the efficient transmission of RGO. The resulting aerogel not only effectively integrates the gas sensing of Eu(TPyP)(Pc) and good conductivity of RGO, but also exhibits a prominent synergy effect. Figure 4 shows the microstructure, responses, and working mechanism of the RGO/Eu(TPyP)(Pc) sensor. A good linear ratio between signal response and target gas concentration is achieved in the range of 0.5–20 and 20–100 ppm.

### 3.4. 3D SnO_2_/RGOH

**Figure 5:**
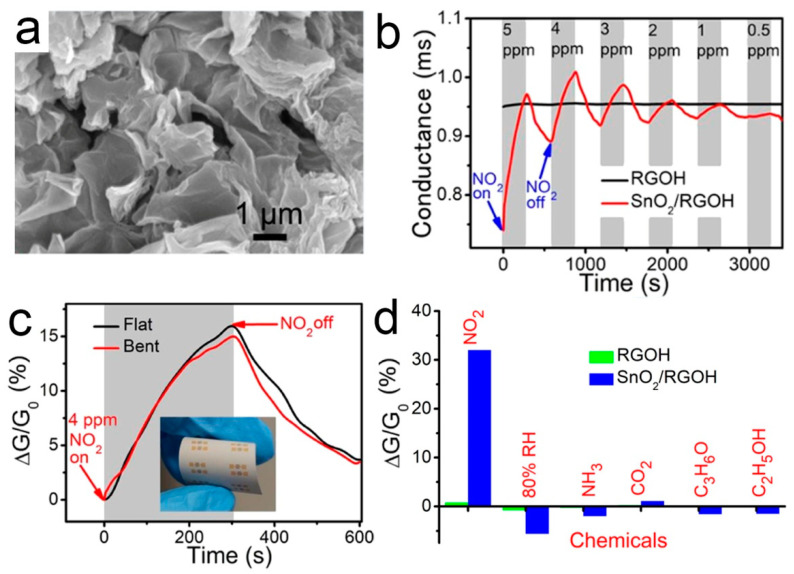
(**a**) SEM image of RGO/Eu(TPyP)(Pc); (**b**) Photograph of RGO/Eu(TPyP)(Pc); inset: water contact angle. (**c**) Dynamic responses of RGO/Eu(TPyP)(Pc) to NO_2_; (**d**) Response-recovery time of RGO/Eu(TPyP)(Pc) to 20 ppm NO_2_; (**e**) Working mechanism of the RGO/Eu(TPyP)(Pc) sensor [88].

Many studies have been devoted to improving the gas sensitivity by immobilizing SnO_2_ crystals on RGO [103,104]. Li and co-workers used SnO_2_ nanocrystals supported by the 3D mesoporous graphene aerogels to detect NO_2_ gas at low temperature [84]. Wu et al. [85] reported a facile preparation of SnO_2_-modified graphene hydrogel (SnO_2_/RGOH) via the one-step hydrothermal method. 3D SnO_2_/RGOH was synthesized directly from Sn^2+^ and GO precursors without any surfactant. The results show that it is feasible to optimize the gas sensing performance by combining reasonable material hybridization, 3D structure, and temperature modulation. The SnO_2_/RGO hybrid showed a high sensitivity to NO_2_ [105]. The improved sensitivity is due to the agglomeration of SnO_2_ nanoparticles and the formation of *p*-*n* heterojunction at the interface between SnO_2_ and RGO [106]. The *p*-*n* heterojunction formed at the interface of RGOH and SnO_2_ promotes the charge transfer. A micro-heater is integrated on the other side of the substrate to increase the substrate temperature locally, so as to suppress the interference of humidity in NO_2_ sensing. Figure 5 shows the microstructure, gas responses, and selectivity of the flexible SnO_2_/RGOH sensor. When exposed to 0.5–5 ppm NO_2_, SnO_2_/RGOH showed an immediately increased conductivity.

### 3.5. 3D Porous B- and N-Doped RGOH

**Figure 6:**
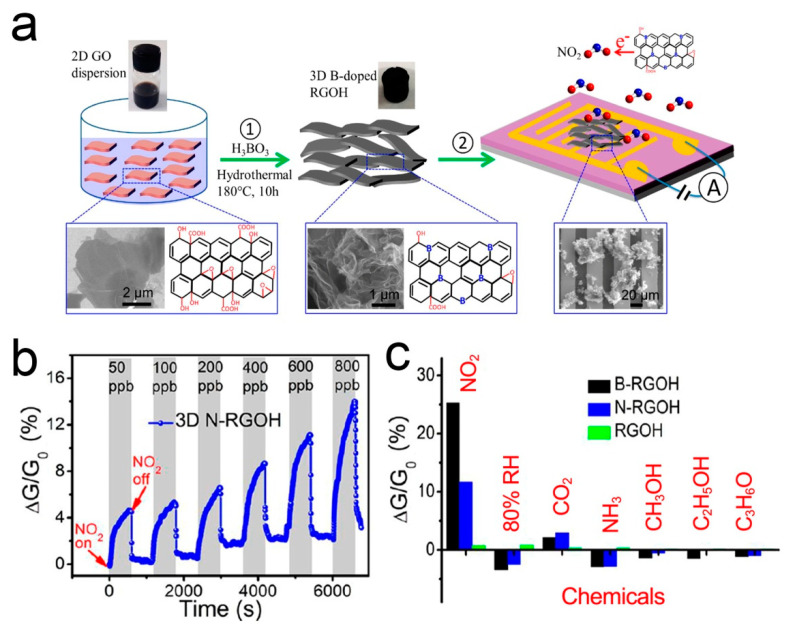
(**a**) Fabrication process of B-RGOH sensors. (**b**) Responses of B- and N-RGOH sensors to NO_2_. (**c**) Responses of B- and N-RGOH sensors to different vapors [87].

Recent studies have shown that doping is a feasible strategy to adjust the physical, electronic, and chemical properties of graphene by creating a band gap [107]. Under the stable adsorption configuration, different elements in graphene can exhibit different gas sensing behaviors due to the different adsorption energy and the distance between doped atoms and gas molecules [108,109,110]. B- and N-doping can improve the selectivity of the graphene-based NO_2_ sensor [111,112,113,114]. Wu et al. [87] reported a 3D porous B- and N-doped RGOH chemical resistor. B- and N-RGOH were synthesized using a hydrothermal self-assembly method with the aid of boric acid (H_3_BO_3_) and dicyandiamide (C_2_H_4_N_4_). Figure 6 shows the fabrication process, microstructures, and gas responses of the sensors. It shows that the combination of 3D hierarchical structure and the doping of B- and N-heteroatoms can significantly improve the sensing performance. The response of B- and N-RGOH to NO_2_ is more than one order of magnitude higher than that of RGOH. It is worth noting that the response of B- and N-RGOH sensors varies almost linearly with the concentration of NO_2_.

## 4. NH_3_ Gas Sensors

The ammonia (NH_3_) sensor is indispensable in many industries and daily life. However, due to its complex preparation process, strict environmental requirements, and desorption of residual ammonia molecules, the production cost is high, which hinders the market acceptance [115,116,117]. The ammonia sensor might exhibit a reduced response during the recovery process in the co-presence of ammonia and ethanol, due to the result of multistep gas adsorption and desorption processes on material surface [115]. In general, the ammonia sensing behavior of the semi-conductive graphene-based sensors is negative to that of NO_2_ sensing, because NH_3_ is the reductive gas and NO_2_ is the oxidative gas [116,117]. For the same graphene-based sensors, however, the sensing performance toward NH_3_ is mostly reported as inferior to that toward NO_2_, for example, in 2020, Wu et al. [89] reported a green synthesis method of 3D chemically functionalized graphene for the high-performance detection of NH_3_ and NO_2_ at room temperature. They found that the LOD of NH_3_ and NO_2_ were 500 and 100 ppb, respectively.

### 4.1. 3D Graphene

**Figure 7:**
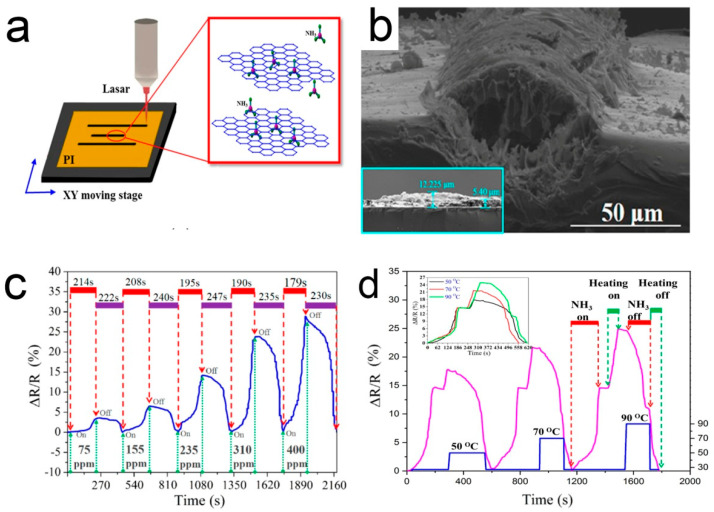
(**a**) A diagram of laser direct writing; (**b**) SEM side view image of the laser-irradiated PI; (**c**) The real-time response/recovery behaviors of the sensor; (**d**) The normalized real-time response/recovery behaviors of the sensor [121].

Generally, when a sensor is recovered at room temperature, the gas molecules cannot be completely desorbed, resulting in poor stability and long recovery time [118]. The desorption of ammonia can be promoted using the infrared light source. This is attributed to the generation of charge carriers by absorbing infrared light [119]. Most of the others used heating to accelerate the desorption process, based on the principle of thermally excited gas molecules [120]. Wu et al. [121] used laser direct writing to fabricate three parallel porous 3D graphene lines on a polyimide (PI) tape to simply construct an ammonia gas sensor. In this study, the ammonia sensor is located in the middle and the two sides are used as heaters. Voltage can be applied to the heater to promote desorption, as shown in Figure 7. The recovery time is relatively stable with the increase of the number of cycles, indicating that the residual ammonia molecules in the element are almost fully desorbed.

### 4.2. 3D RGO/PPy

**Figure 8:**
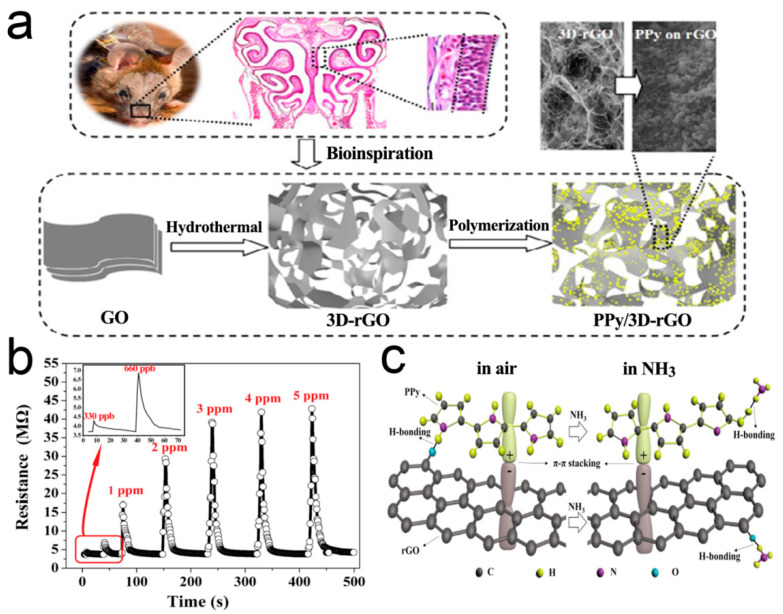
(**a**) Fabrication process for the 3D crumpled PPy/3D-rGO nanocomposite; (**b**) Dynamic response of the PPy/3D-RGO sensor to NH_3;_ (**c**) Schematic illustration depicting the interaction of NH_3_ with PPy/3D-RGO [129].

Recent studies have witnessed the significant progress of gas sensors based on polypyrrole (PPy) conducting polymers [122,123,124,125]. However, the serious agglomeration of the nanoparticles leads to a significant decrease in the visible active surface of gas molecules, which leads to the decrease of response sensitivity [126,127,128]. Qin et al. [129] used 3D RGO as a 3D skeleton support for the sensitive PPy nanoparticles. It has made an important contribution to improve the conductivity, dynamic performance, and sensing response, as shown in Figure 8. Highly dispersed and low agglomerated PPy nanoparticles are uniformly distributed on the pore walls of 3D RGO. The response to NH_3_ can rapidly reach sub-ppm level, 4–5 times more sensitive than that of pure PPy. The sensor has perfect stability.

### 4.3. PANI/CuO@3D-NGF

CuO nanoparticles are suitable for the gas detection due to their narrow band gap and adjustable morphology [130,131,132]. However, the problem is that they can only operate effectively at high temperatures. To improve this problem, nanoparticles can be incorporated into porous materials to construct the functional composites [102,133]. Tabar et al. [134] prepared a chemical-resistant ammonia sensor based on polyaniline (PANI)/CuO nanoparticles, supported on a 3D N-doped graphene framework (NGF). The sensor has an excellent response to 100 ppm NH_3_, with an outstanding LOD down to 50 ppb. The average response time is 30 s at room temperature. It is not sensitive to other gases and has good selectivity to NH_3_. The excellent sensing performance was attributed to 3D interconnected porous structure, remarkable enhancement of charge carriers, and modified π-interactions between molecules [134].

## 5. Phenol Gas Sensors

Phenol is the natural component of many substances and can be emitted from the combustion of fossil fuels and tobacco [20]. It is also present in animal wastes and decomposing organic material. More importantly, it is a chemical product produced at a rate of about 6 million ton/year worldwide [20]. The manufacture and transportation of phenol as well as its many uses may lead to worker exposures to this substance with health risks [135]. In 2013, Liu et al. [136] prepared an electrochemical sensor using assembled 3D graphene as the electrode. The LOD of the phenol sensor mediated by tyrosine was successfully achieved down to 50 ppm. In 2016, Guo et al. [137] reported RGO/metal-oxide *p*-*n* heterojunction aerogels as efficient 3D sensing frameworks for phenol detection. Upon the detection of phenol at room temperature, the sensor has good sensitivity, repeatability, and stability. The linear relationship is in the range of 10–80 ppb. Compared with the detection results of ethanol, toluene, and methanol, the gas sensor based on RGO/SnO_2_ composite aerogel has much higher sensitivity to phenol with the LOD down to 5 ppb. In the same year, Gao et al. [138] reported highly sensitive electrocatalytic determination of phenols based on coupled cMWCNT/cyclodextrin edge-functionalized graphene composite. The sensor has excellent performance towards trace detection of three typical phenols (4-aminophenol, 4-AP; 4-chlorophenol, 4-CP; 4-nitrophenol, 4-NP). Under optimal conditions, the current responses of 4-AP, 4-CP, and 4-NP are linear to concentrations over two different ranges, with the LODs of 0.019, 0.017, and 0.027 ppm (S/N = 3), respectively. In 2019, Qi et al. [139] reported a facile synthesis of 3D S/N co-doped graphene derived from GO hydrogel. This newly fabricated sensor was used in the simultaneous detection of catechol and hydroquinone, with the LODs of 0.28 and 0.15 ppm, respectively.

## 6. Conclusions

Compared with the 2D graphene nanosheets, the signal transduction of 3D graphene frameworks are more than ten times higher in most gas detection, due to the increase of the interaction surface area and the number of active adsorption sites [89]. In addition to the original 2D structural properties of graphene, its 3D porous frameworks are more favorable. It is good for adsorption and diffusion of gas molecules. In general, the LOD can be improved from ppm level to several ppb level. This has a great impact on the prevention of toxic and harmful air pollution to the atmosphere.

In addition, 3D graphene has a strong mechanical strength and a high temperature resistance, capable of using in harsh environments. These properties are very promising for practical application. It is a potential research direction to combine 3D graphene with flexible substrate to make wearable flexible sensors.

However, 3D graphene is still limited in wide applications due to its limited gas sensing types. The main toxic pollutants in the air are NO_x_ (main NO_2_), NH_3_, CO, CH_2_O, and phenol. Compared with traditional metal oxide sensing materials, graphene is superior due to the much lower operating temperature and much lower resistance. It requires a low energy consumption in operation [83,87,115]. As shown in Table 2, traditional semiconductor-based gas sensors generally work at 200–400 °C [98,140,141,142]. Some commercial gas sensors could work well at room temperature, but with some performance loss of the LOD [143,144]. Compared to individual MoS_2_, SnS_2_, organic compounds (e.g., PPy), and semiconductor metal oxides (e.g., SnO_2_), the sensors based on 3D graphene composites could see an increase of the sensitivity.

At present, 3D graphene is mainly sensitive to NO_2_ and NH_3_, and more gas sensors need to be discovered. The adsorption and desorption of 3D graphene need to be accelerated so as to reduce the response and recovery times of the sensor. Finally, 3D graphene sensors are still in the stage of laboratory investigation, and more work is needed to put forward these developments to the commercialization stage.

## Figures and Tables

**Table 1:** Gas sensitivities of 3D graphene toward NO_2_.

3D Graphene	Temp. (°C)	*C*_NO2_ (ppm)	Response	S_3D_ (ppm^−1^)	S_3D_/S_2D_	Recovery Time (s)	LOD (ppb)	Year	Ref.
Superhydrophobic 3D RGO	113	1	∆G/G_0_ = 23.5%	23.5%	-	169	9.1	2018	[82]
3D S-RGOH	RT	2	∆R/R_0_ = 22.5%	8.7%	118.6	11	4.1	2017	[83]
3D RGO-SnO_2_	55	100	∆R/R_0_ = 6.5%	0.1%	-	500	2000	2015	[84]
3D SnO_2_/RGOH	RT	5	∆G/G_0_ = 32%	4.3%	62.9	260	2.8	2020	[85]
3D MoS_2_/RGO	80	10	∆I/I_0_ = 2483%	248%	>250	30	27.9	2019	[52]
3D SnS_2_/RGO	RT	8	∆G/G_0_ = 49.8%	6.1%	22.6	76	8.7	2020	[86]
3D N-RGOH	RT	0.8	∆G/G_0_ = 11.7%	8.7%	18	10	14	2019	[87]
3D B-RGOH	RT	0.8	∆G/G_0_ = 25.3%	20%	38.9	90	9	2019	[87]
3D RGO/Eu(TPyP) (Pc)	RT	20	∆I/I_0_ = 12%	0.6%	2	828	80	2020	[88]
VC-Funct. RGOH	RT	10	∆G/G_0_ = 36.3%	3.6%	10	300	100	2020	[89]

Note: The response, in the form of relative change of resistance (∆R/R_0_) or conductance (∆G/G_0_), was tested at a set temperature and a set concentration of NO_2_ (C_NO2_). S_3D_ and S_2D_ are the sensitivities (responses per ppm) of 3D and 2D graphene, respectively. LOD = limit of detection.

**Table 2:** Sensing performance comparison of 3D graphene and other semiconductor materials including commercial sensors.

Sensing Materials	Gas	Temp. (°C)	*C_gas_* (ppm)	Response	S (ppm^−1^)	Recovery Time (s)	LOD (ppb)	Year	Ref.
MoS_2_	NO_2_	80	10	∆I/I_0_ = 120%	12%	-	-	2019	[52]
3D MoS_2_/RGO	NO_2_	80	10	∆I/I_0_ = 2483%	248%	30	27.9	2019	[52]
SnS_2_	NO_2_	160	8	∆G/G_0_ = 28%	3.5%	140	20–30	2015	[98]
3D SnS_2_/RGO	NO_2_	RT	8	∆G/G_0_ = 49.8%	6.1%	76	8.7	2020	[86]
SnO_2_	NO_2_	400	5	∆R/R_0_ = 10%	2%	720	-	2016	[142]
3D SnO_2_/RGOH	NO_2_	RT	5	∆G/G_0_ = 32%	4.3%	260	2.8	2020	[85]
PPy	NH_3_	RT	3	R_g_/R_a_ = 2%	0.6%	-	-	2019	[129]
3D RGO/PPy	NH_3_	RT	3	R_g_/R_a_ = 10.5%	10.5%	25	-	2019	[129]
TypeNO_2_/S-100	NO_2_	RT	1	−370 ± 70 (nA·ppm^−1^)		<60	<200	Comm.	[143]
TypeNH_3_/SR-200	NH_3_	RT	1	90 ± 18 (nA·ppm^−1^)		<50	<600	Comm.	[144]
